# Supplementary material for: Multi-domain O-GlcNAcase structures reveal allosteric regulatory mechanisms
Source: Nat Commun. 2025 Oct 3;16:8828. doi: 10.1038/s41467-025-63893-2 (PMC12494797; doi:10.1038/s41467-025-63893-2)

Supplementary Information for "Multi-domain O-GlcNAcase structures reveal allosteric regulatory mechanisms"

Sara Basse Hansen[1,+], Sergio G. Bartual[1,+], Huijie Yuan[1,2], Olawale G. Raimi[2], Andrii Gorelik[2,5], Andrew T. Ferenbach[1], Kristian Lytje[3,4], Jan Skov Pedersen[3,4], Taner Drace[1,3], Thomas Boesen[1,3], and Daan M. F. van Aalten[1,2,*]

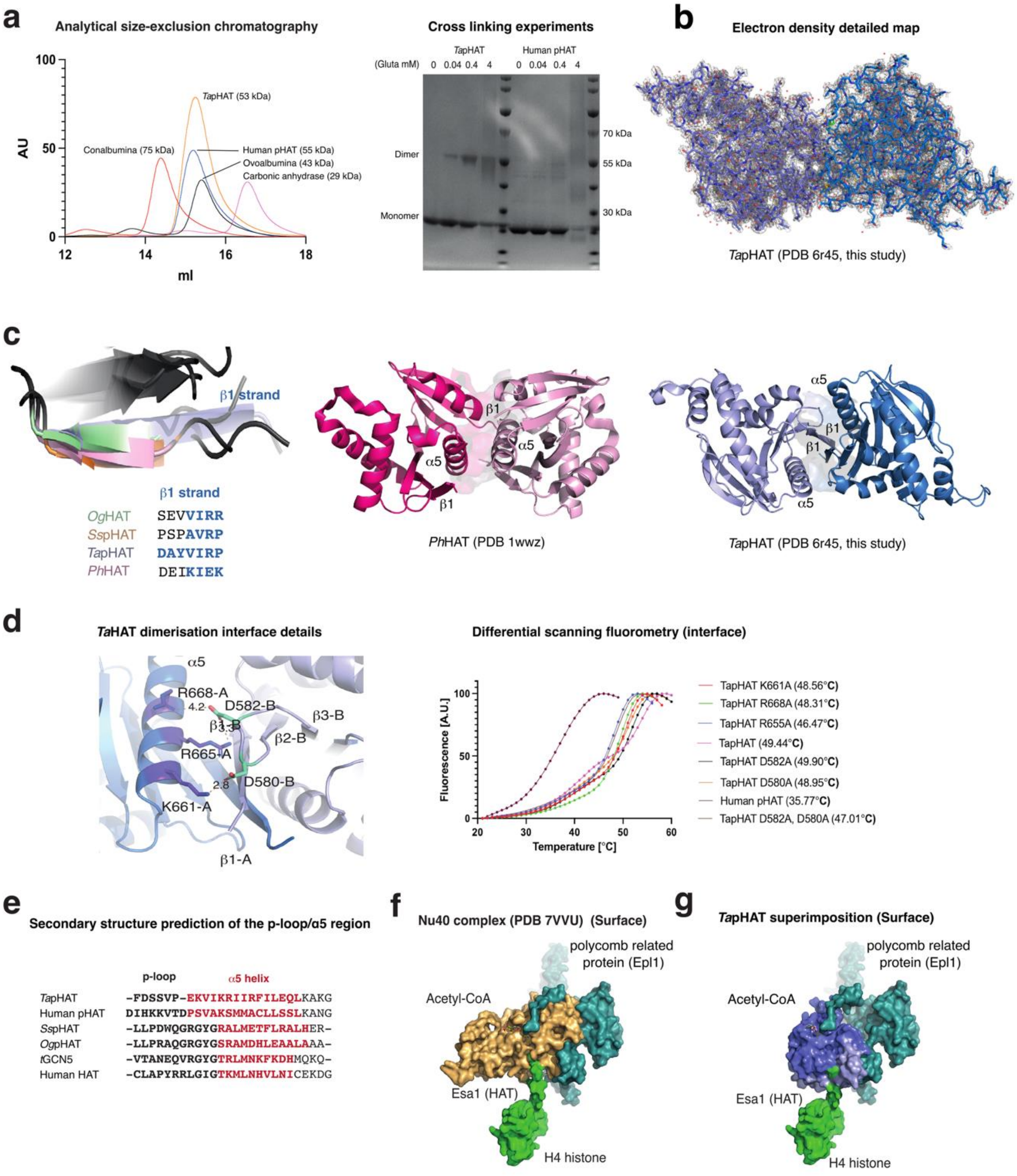

a
Analytical size-exclusion chromatography
AU
ml
100
50
0
12
14
16
18
TapHAT (53 kDa)
Conalbumina (75 kDa)
Human pHAT (55 kDa)
Ovoalbumina (43 kDa)
Carbonic anhydrase (29 kDa)
Cross linking experiments
TapHAT
Human pHAT
(Gluta mM)
0 0.04 0.4 4
0 0.04 0.4 4
Dimer
Monomer
70 kDa
55 kDa
30 kDa
b
Electron density detailed map
TapHAT (PDB 6r45, this study)
c
β1 strand
β1 strand
OgHAT SEVVIRR
SspHAT PSPAVRP
TapHAT DAYVIRP
PhHAT DEIKIEK
β1
α5
α5
β1
PhHAT (PDB 1wwz)
α5
β1
β1
α5
TapHAT (PDB 6r45, this study)
d
TaHAT dimerisation interface details
α5
R668-A
4.2
D582-B
β1-B
3.3
β3-B
β2-B
R665-A
2.8
D580-B
K661-A
β1-A
Differential scanning fluorometry (interface)
Fluorescence [A.U.]
Temperature [°C]
100
50
0
20
30
40
50
60
TapHAT K661A (48.56°C)
TapHAT R668A (48.31°C)
TapHAT R655A (46.47°C)
TapHAT (49.44°C)
TapHAT D582A (49.90°C)
TapHAT D580A (48.95°C)
Human pHAT (35.77°C)
TapHAT D582A, D580A (47.01°C)
e
Secondary structure prediction of the p-loop/α5 region
p-loop
α5 helix
TapHAT -FDSSVP-EKVIKRIIRFILEQLKAKG
Human pHAT DIHKKVTDPSVAKSMMACLLSSLKANG
SspHAT -LLPDWQGRGYGRALMETFLRALHER-
OgpHAT -LLPRAQGRGYGSRAMDHLEAALAAA-
tGCN5 -VTANEQVRGYGTRLMNKFKDHMQKQ-
Human HAT -CLAPYRRLGIGTKMLNHVLNICEKDG
f
Nu40 complex (PDB 7VVU) (Surface)
polycomb related protein (Epl1)
Acetyl-CoA
Esa1 (HAT)
H4 histone
g
TapHAT superimposition (Surface)
polycomb related protein (Epl1)
Acetyl-CoA
Esa1 (HAT)
H4 histone

Supplementary Figure 1: **Crystal structure of the *Ta*OGA pHAT domain dimer.**
a) Analysis of the oligomeric state of *Trichoplax adhaerens* and human pHAT domains in solution. Left: Analytical size exclusion profiling including known molecular weight standards. Molecular weight of standards and samples is indicated in brackets. Right: Cross-linking experiments of *Ta*pHAT and human pHAT using increasing concentrations of glutaraldehyde from left to right (untreated protein (lane 1) and treated with 0.044 mM (lane 2), 0.44 mM (lane 3) and 4.4 mM glutaraldehyde (lane 4).
b) Electron density (2Fo-Fc) map (grey mesh) calculated for the crystallographic model of the *Ta*pHAT structure are displayed at 1.5 σ contour level. Proteins are represented as a stick model with one monomer coloured in slate and the other in marine.
c) Comparison of the β1 strand length and amino acid composition among the HAT and pHAT domains from the *Pyrococcus horikoshii Archaeota (Ph*HAT*)*[1](pink), the *Oceanicola granulosus* bacterial HAT (*Og*HAT) [2] (green), the *Saccharomyces cerevisiae* pHAT domain (*Ss*pHAT) [3] (orange) and *Ta*pHAT (slate). The right panels depict the dimeric arrangement of the *Ph*HAT (red and pink) and the *Ta*pHAT domain (slate and marine).
d) Hydrogen bonds and electrostatic interactions found in the dimerisation interface. Monomer A is coloured marine, and the α5 helix-interacting residues are in blue magenta. Monomer B is coloured slate, and the interacting residues from the β2- β3 loop are in teal. Differential scanning fluorometry results of the interface residues alanine point mutants plus controls (*Ta*pHAT and human pHAT) are shown on the right-hand side (n=6 independent experiments for each data point).
e) Sequence alignment of the pHAT domain p-loop and α5 regions across evolution.
f) Details of the Nu40 HAT complex elements. Esa1 (wheat), H4 histone (green) and Epl1 (cyan).
g) Details of the superposition of the *Ta*pHAT domain onto the Nu40 HAT complex, highlighting potential steric clashes between the *Ta*pHAT domain and the H4 histone in this specific arrangement. *Ta*pHAT (slate and marine), H4 histone (green) and Epl1 (cyan).

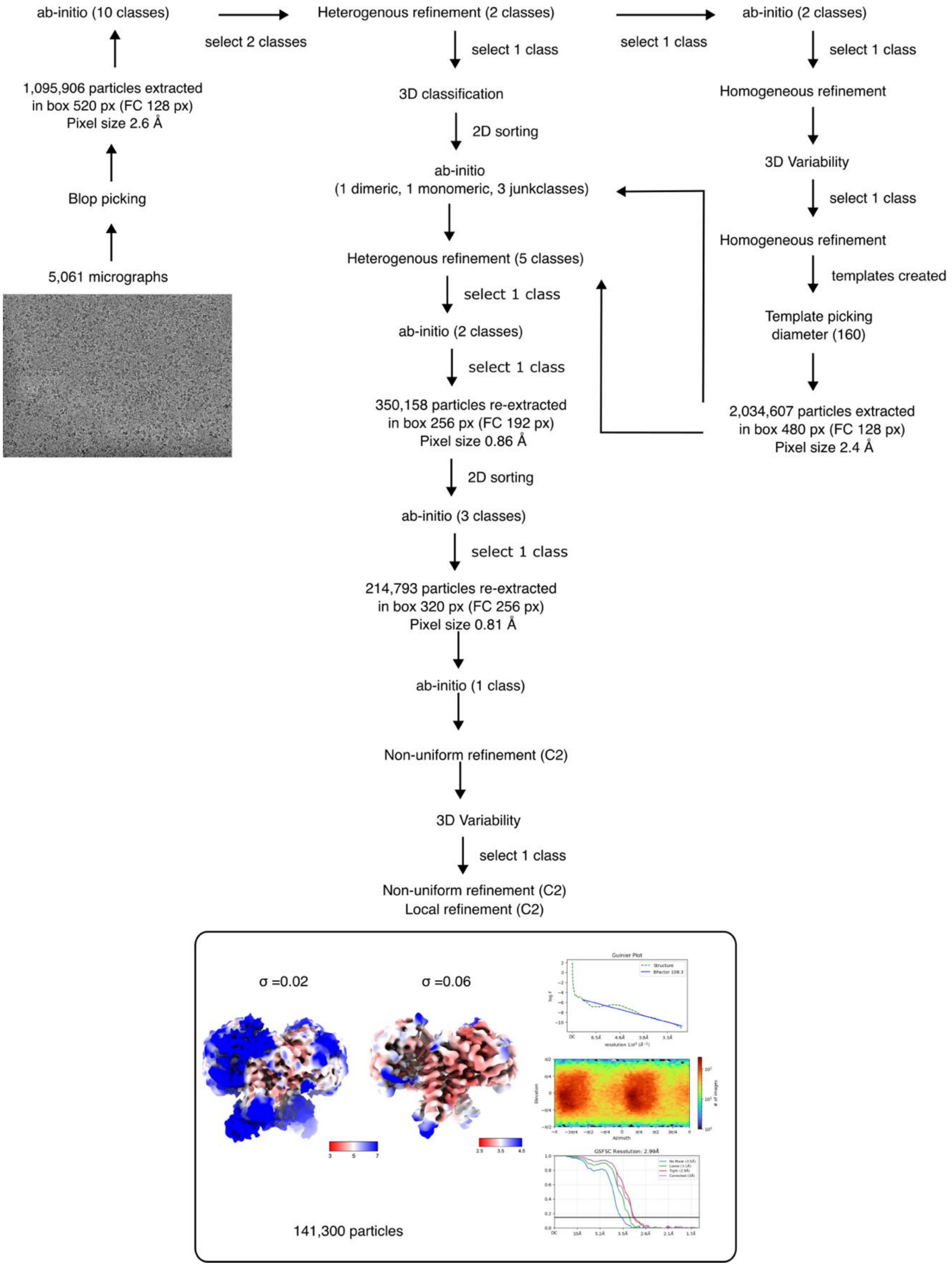

Supplementary Figure 2: Cryo-EM pipeline for *Ta*OGA. Data was processed in CryoSPARC. The final structure from refinement with applied C2 symmetry is shown with its local resolution represented by a various colours. Plots from the final refinement are shown.

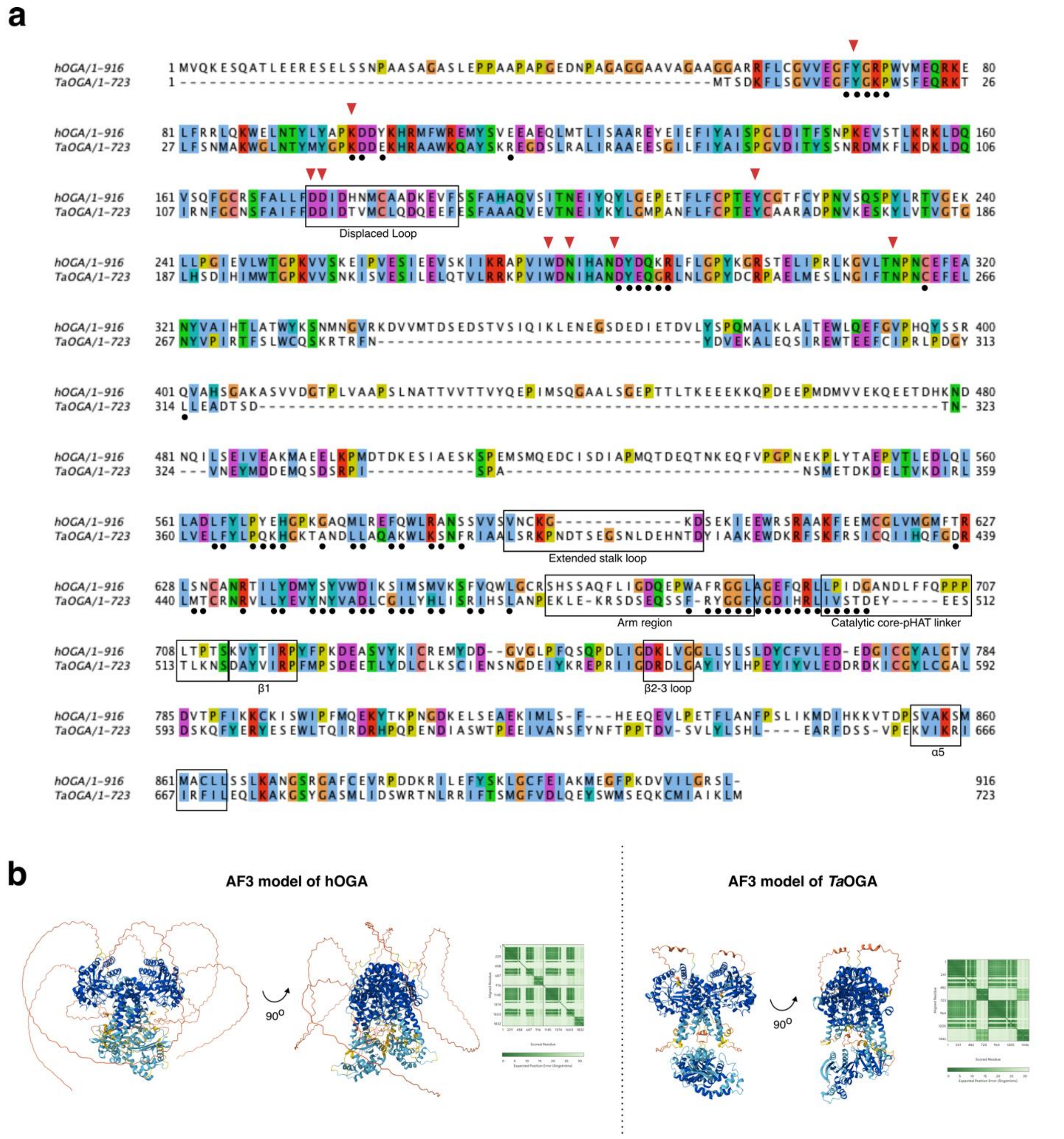

Supplementary Figure 3: Comparison of hOGA and *Ta*OGA.

a) Sequence alignment of hOGA and *Ta*OGA. Red arrows above specific amino acids indicate residues coordinating GlcNAc in the active site, and black circles below specific amino acids indicate the dimer interface for the *Ta*OGA cryo-EM structure, as calculated by PISA. Specific regions mentioned throughout the paper are highlighted in boxes.

b) AlphaFold3 (AF3) models of hOGA and *Ta*OGA coloured according to their pLDDT score. The Expected Position Error is shown as a heatmap for both models

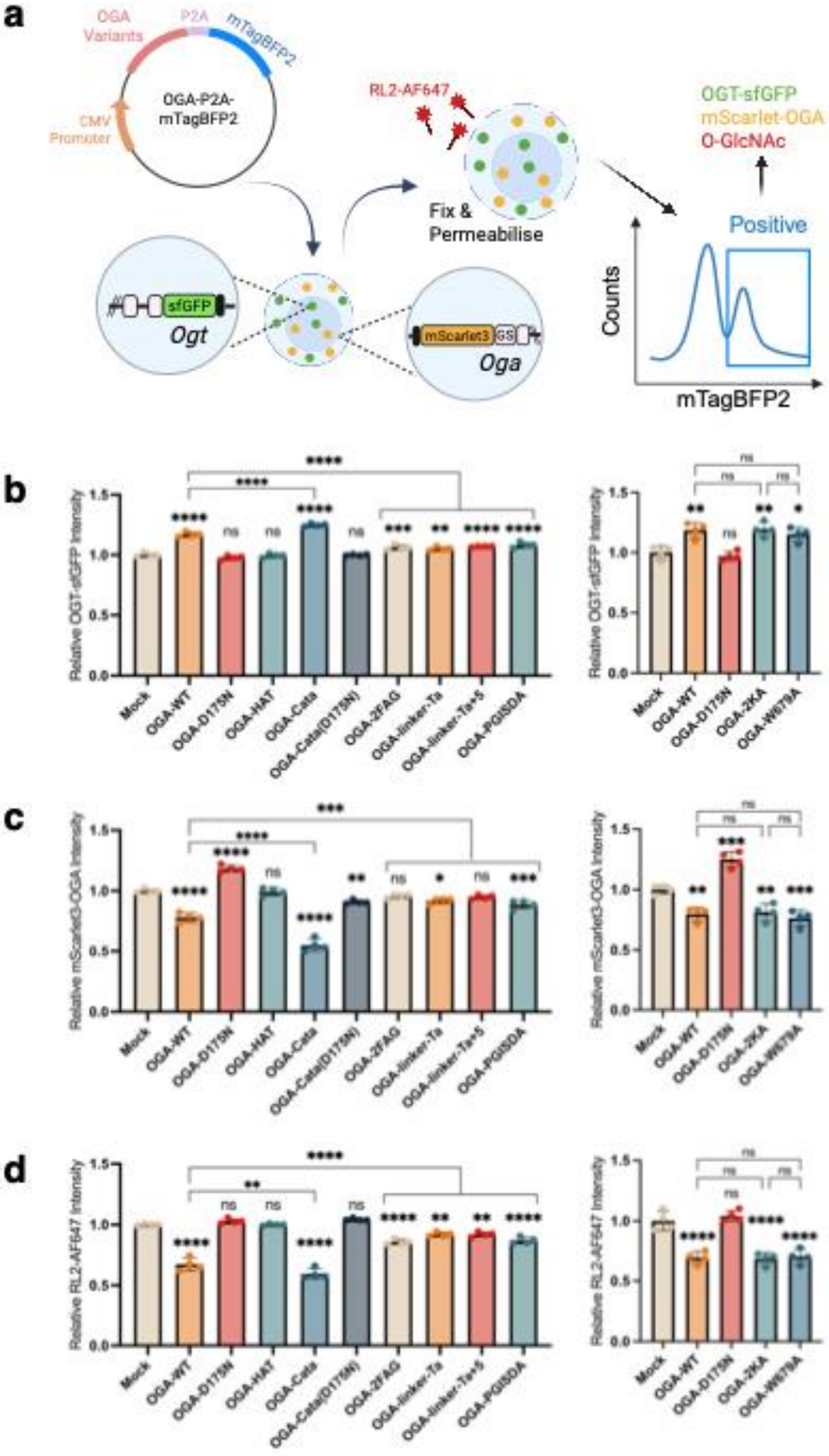
a
OGA Variants
P2A
mTagBFP2
OGA-P2A-mTagBFP2
CMV Promoter
RL2-AF647
Fix & Permeabilise
OGT-sfGFP
mScarlet-OGA
O-GlcNAc
Positive
Counts
mTagBFP2
sfGFP
Ogt
mScarlet3
GS
Oga
b
Relative OGT-sfGFP Intensity
Mock
OGA-WT
OGA-D175N
OGA-HAT
OGA-Cata
OGA-Cata(D175N)
OGA-2FAG
OGA-linker-Ta
OGA-linker-Ta+5
OGA-PGISDA
OGA-2KA
OGA-W679A
c
Relative mScarlet3-OGA Intensity
d
Relative RL2-AF647 Intensity

Supplementary Figure 4: Disruption of O-GlcNAc homeostasis by hOGA variants.

a) Brief outline of the double-fluorescently labelled mESC system to detect changes in O-GlcNAc homeostasis (as measured by changes in endogenous OGT, OGA and global O-GlcNAc levels) as a result of transfecting exogenous mTagBFP2-labelled hOGA variants (see M&M for details)**.** The schematic was adapted from previous work[4]. Created in BioRender. Sevillano quispe, O. (2025) https://BioRender.com/s0yv8qd.

b-d) Panel b shows changes in endogenous OGT-sfGFP levels, panel c shows changes in endogenous mScarlet3-OGA levels, and panel d shows changes in global O-GlcNAc levels. Compared to mTagBFP2 only transfection (Mock), transfection with wild-type OGA (OGA-WT) resulted in increased OGT-sfGFP levels (panel b), decreased mScarlet3-OGA levels (panel c), and a corresponding reduction in global O-GlcNAc levels (panel d). Deletion of the pHAT domain (OGA-Cata) further exacerbated these effects, with a slightly greater increase in OGT levels and more pronounced reductions in both OGA and global O-GlcNAc levels (panels b-d), suggesting an enhanced disruption of O-GlcNAc homeostasis. Introducing the catalytic dead mutation D175N into this construct (OGA-Cata(D175N)) abolished these effects (panels b-d). Transfection with the hOGA pHAT domain alone (OGA-pHAT) had no impact on OGT, OGA, or O-GlcNAc levels.

Substitution of the conserved tryptophan at position 679 with alanine (OGA-W679A), or mutation of Lys597 and Lys599 to alanine (OGA-2KA), did not alter the extent of O-GlcNAc disruption compared to OGA-WT (panels b-d). Additional constructs tested include the double phenylalanine mutant (OGA-2FAG; Phe703Ala and Phe704Gly), OGA-linker-Ta, OGA-linker-Ta+5, and OGA-PGISDA, as referenced in the main text (panels b-d). Statistical analysis was performed using ordinary one-way ANOVA (n = 4 biologically independent replicates, each transfected on separate days using cells of different passage numbers). Statistical significance between Mock and each variant is indicated above the respective bars. Selected pairwise comparisons are shown with connecting lines, and adjusted *p*-values are represented as follows: ns ($p > 0.05$), * ($p < 0.05$), *** ($p < 0.001$), **** ($p < 0.0001$). Error bars represent mean ± SD.

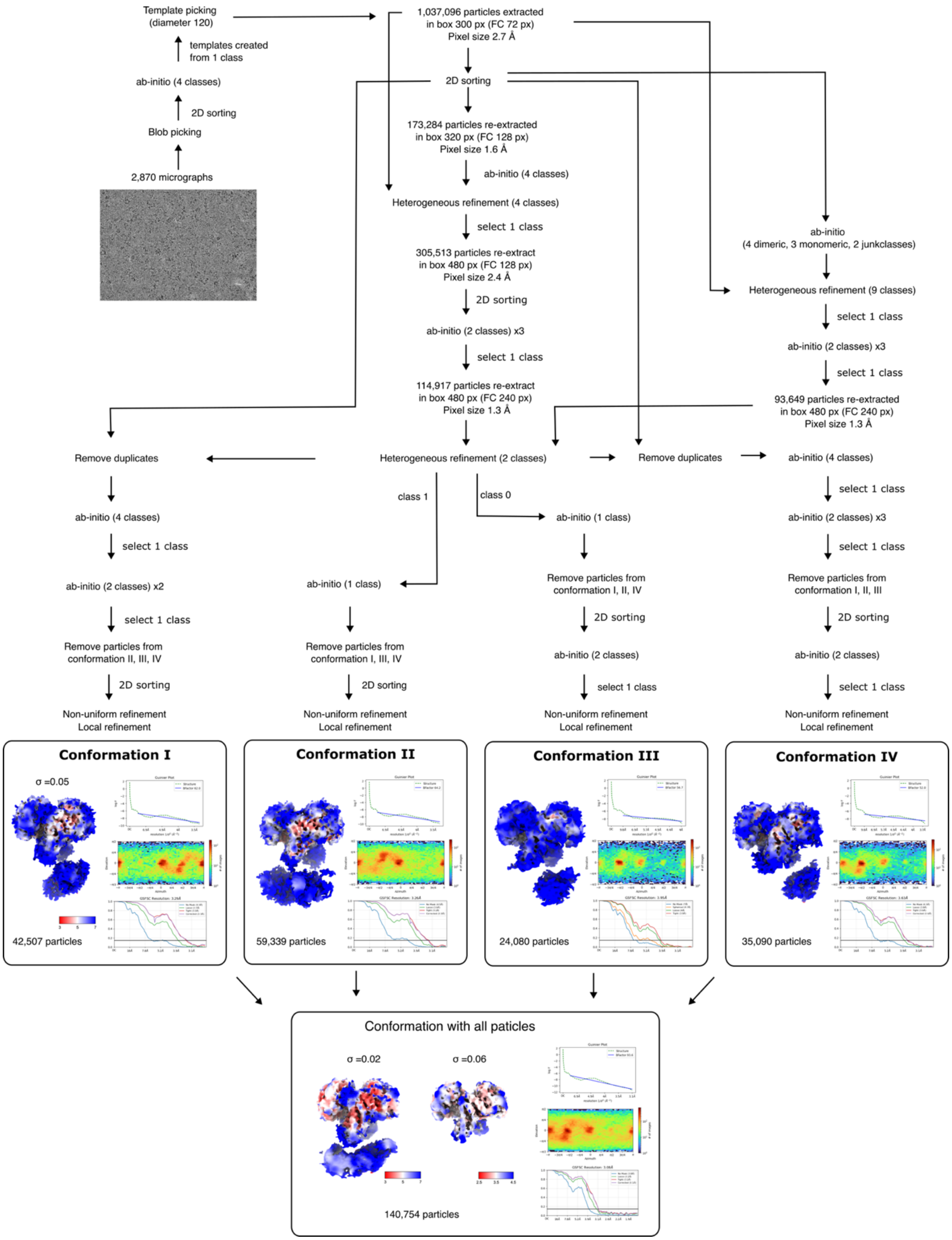

Supplementary Figure 5: Cryo-EM pipeline for hOGA. Data was processed in CryoSPARC. The final structure from refinement is shown with its local resolution represented by a various colours. Plots from the final refinement are shown.

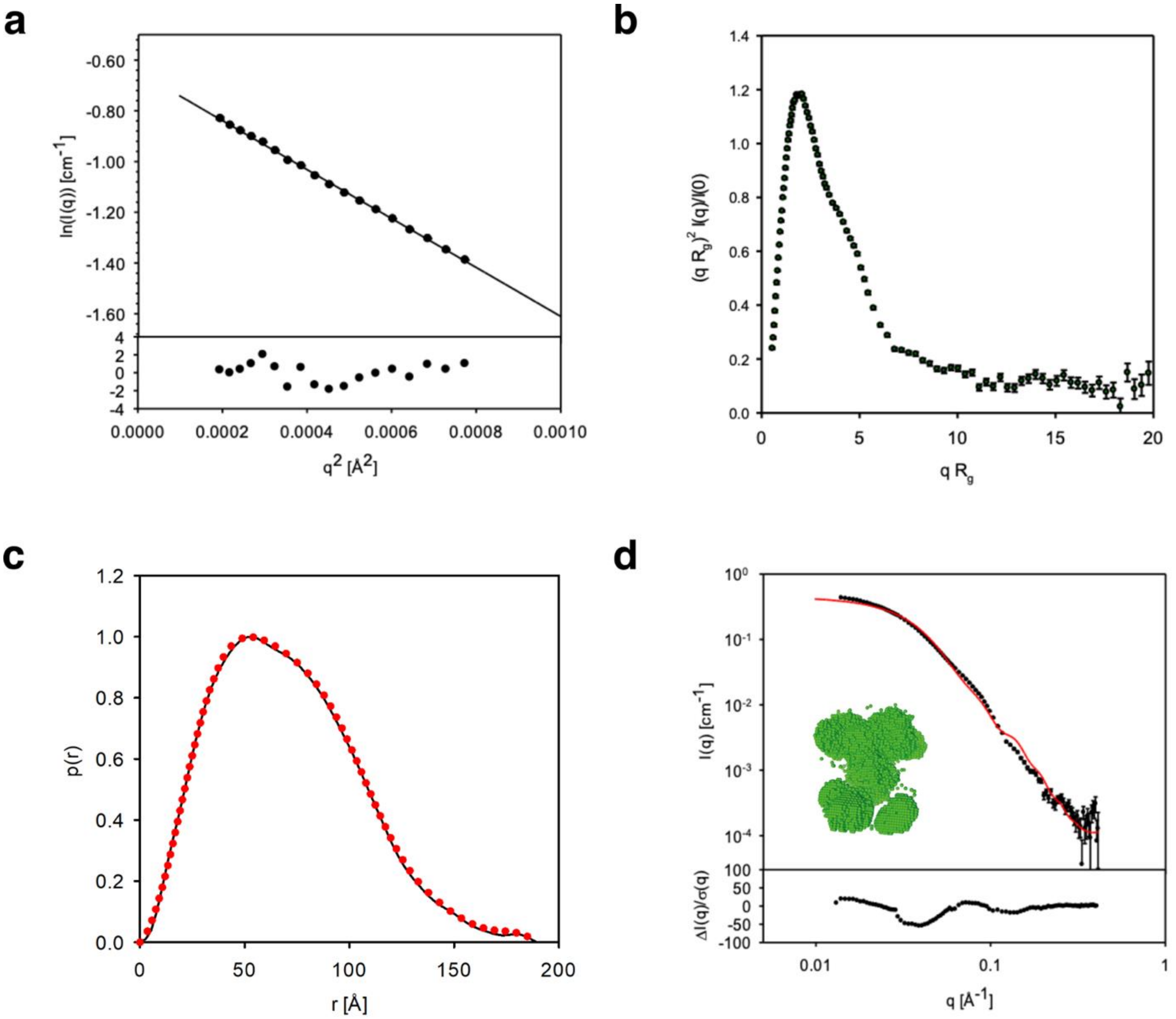

Supplementary Fig. 6: **SAXS data summary figure for hOGA.**

a) Guinier fit (top) and fit residuals (bottom).

b) Dimensionless Kratky plot. Dashed lines show where a globular system would peak.

c) *p(r)* function, normalised by the maximum value. The function from BIFT is shown as full curve and the one from AutoGNOM is shown as dotted curve.

d) Fit to the SAXS data for the best models from rigid-body optimization. The black points are the SAXS data for hOGA. The red curve is the model fit. Real space models from SAXS generated for the cryo-EM map of hOGA is show as green spheres.

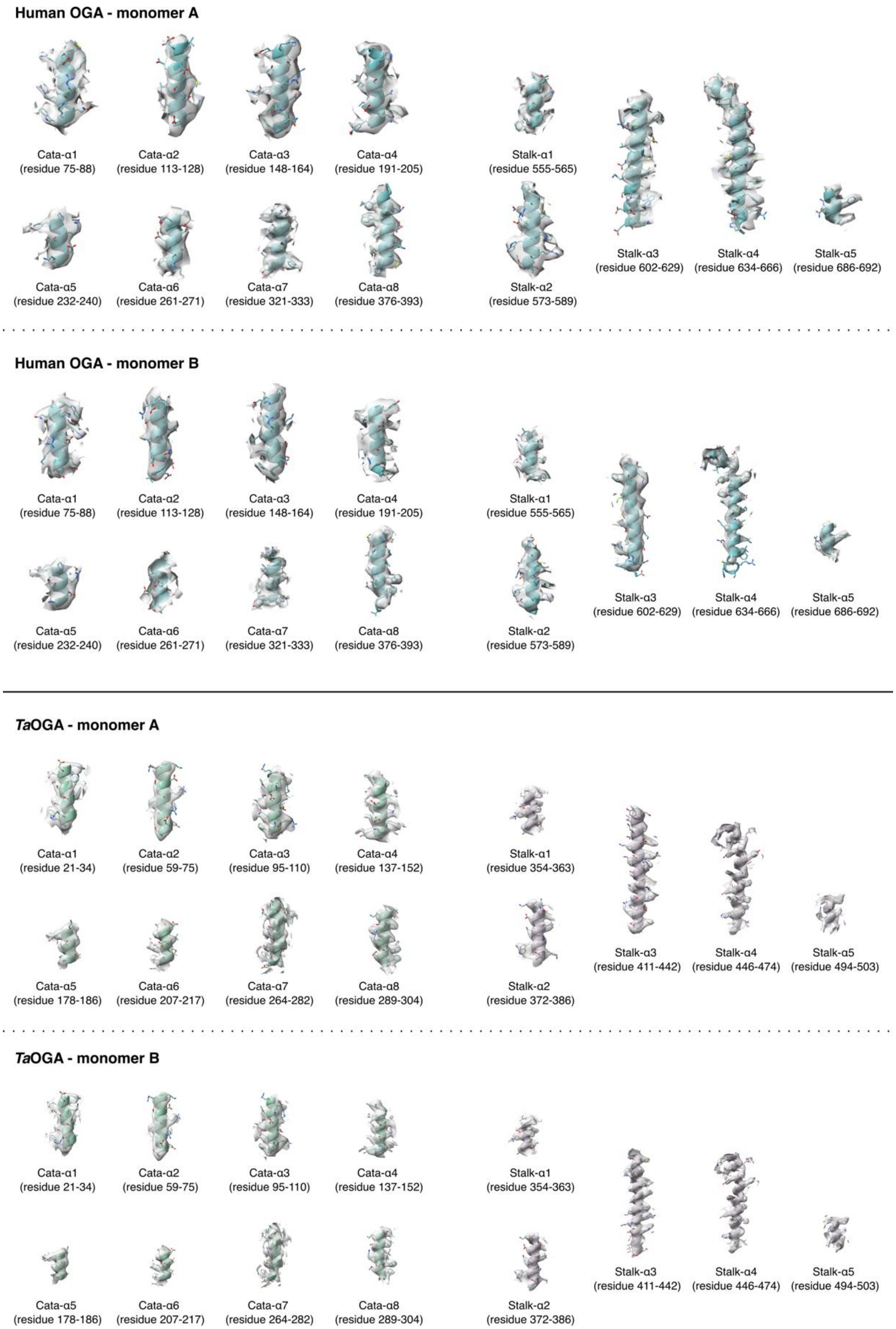

Supplementary Figure 7: Model-to-map fit. Individual helix placement inside map to evaluate the quality of fit. Helices are represented both as cartoons and sticks. Human OGA is coloured in teal, and the catalytic domain is green and the stalk domain pink for *Ta*OGA.

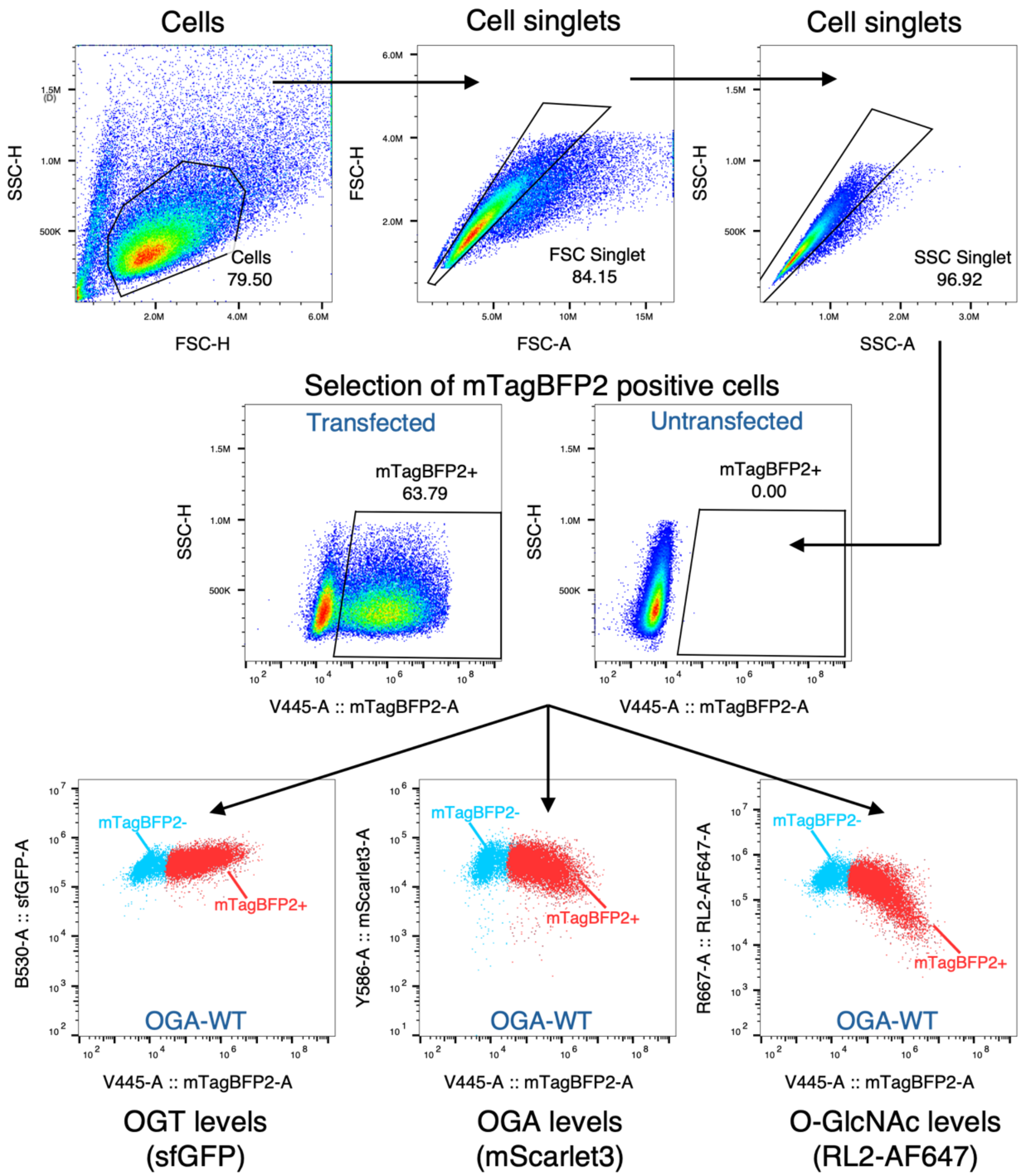

Supplementary Figure 8: **Gating strategy for OGT-sfGFP/mScarlet3-OGA mESCs fluorescence detection.** Cells were analysed using forward scatter height (FSC-H) and side scatter height (SSC-H) to distinguish cells from debris. Gated cells were then assessed using FSC-A vs. FSC-H and SSC-A vs. SSC-H plots to identify singlet populations. Gated singlet cells were subsequently analysed based on mTagBFP2 fluorescence (mTagBFP2-A) to identify transfected, mTagBFP2-positive cells. Endogenous OGT and OGA protein levels, as well as global O-GlcNAc levels, were quantified by measuring the median fluorescence intensity of OGT-sfGFP, mScarlet3-OGA, and RL2-Alexa Fluor 647, respectively, within the mTagBFP2-positive population. These values were then normalised to the corresponding fluorescence intensities in mTagBFP2-negative cells from the same sample and used for statistical analysis.

Supplementary Table 1: Diffraction data and refinement statistics for the *Ta*pHAT crystal structure

**Measurement**	***Ta*pHAT**
Resolution	59.59 - 1.784 (1.848 - 1.784)
Space group	P1
Unit cell<br>a (Å), α<br>b (Å), β<br>c (Å), γ	 43.25, 87.52<br>43.52, 85.24<br>59.84, 87.58
No. of reflections	41343
No. of unique reflections	36731(3663)
I/σ (I)	8.0 (3.0)
Completeness (%)	0.89
Redundancy	1.6
R_{merge}	0.07
$CC_{1/2}$	0.99 (0.930)
RMSD from ideal geometry<br>Bond distance (Å)<br>Bond angle (°)	 1.32<br>0.008
R_{work} (%)	0.202 (0.260)
R_{free} (%)	0.232 (0.296)
No. of residues	410
No. of water mol	170
B factors (Å^2)<br>Overall<br>Protein<br>Ligand<br>solvent	 23.5<br>23.4<br>25.2<br>26.3
PDB ID	6R45

Values in parenthesis are for the highest resolution shell. All measured data were included in the refinement.

Supplementary Table 2: SAXS data summary

	H5.RSR (hOGA)
Guinier R_g [Å]	54.1 +/- 0.3
Guinier $I(0)$ [cm^{-1}]	0.53 +/- 0.002
M.W. (V_p) [kDa]	278
M.W. (V_c) [kDa]	285
GNOM D_{max} [Å]	196.0
GNOM R_g [Å]	55.3 +/- 0.2
GNOM $I(0)$ [cm^{-1}]	0.527 +/- 0.002
BIFT D_{max} []	189 +/- 6
BIFT Rg [Å]	54.8 +/- 0.2
BIFT $I(0)$ [cm^{-1}]	0.525 +/- 0.001

Supplementary Table 3: Data collection and refinement statistics for cryo-EM analysis

**Data collection and processing**	***Ta*OGA**	**hOGA**
Magnification	130,000x	130,000x
Voltage (kV)	300	300
Microscope	Titan Krios G3i	Titan Krios G3i
Camera	Gatan K3	Gatan K3
Physical pixel size (Å/pix)	0.647	0.647
Electron exposure (e-/Å^2)	59.9	60.0
Defocus range (μm)	-0.8 to -2.0	-0.8 to -2.0
Total number of movies	5,061	2,870
Initial particles	1,095,906	1,037,096
Final particles	141,300	140,754
Symmetry imposed	C2	C1
Map resolution (Å)	2.99	3.08
FSC threshold	0.143	0.143
**Refinement**		
Initial model used	AF model	AF model
Chains	2	2
Atoms	6944	7326
Residues	848	898
Ligands	0	0
Average B factor (Å^2)	141.26	143.80
Bond length RMSD (Å)	0.004	0.005
Bond angle RMSD (°)	0.886	0.882
MolProbity score	2.37	2.14
Clashscore	3.19	2.48
Ramachandran outliers (%)	0.00	0.00
Ramachandran allowed (%)	7.25	5.10
Ramachandran favored (%)	92.75	94.90
PDB code	9QEP	9QEN
EMDB code	EMD-53082	EMD-53081

Supplementary Table 4: Primers used to introduce mutations in the predicted *Ta*pHAT dimeric interphase

54H_K661A_fwd	CTAGATTTGATTCATCTGTTCCGGAAgcGGTCATAAAAAGAATAATTCGATTTATAC
54H_K661A_rev	GTATAAATCGAATTATTCTTTTTATGACCgcTTCCGGAACAGATGAATCAAATCTAG
54H_R665A_fwd	CATCTGTTCCGGAAAAGGTCATAAAAgcAATAATTCGATTTATACTTGAACAATTG
54H_R665A_rev	CAATTGTTCAAGTATAAATCGAATTATTgcTTTTATGACCTTTTCCGGAACAGATG
54H_R668A_fwd	CCGGAAAAGGTCATAAAAAGAATAATTgcATTTATACTTGAACAATTGAAAGCTAAAGGG
54H_R668A_rev	CCCTTTAGCTTTCAATTGTTCAAGTATAAATgcAATTATTCTTTTTATGACCTTTTCCGG
54H_trip_fwd	GATTCATCTGTTCCGGAAgcGGTCATAAAAgcAATAATTgcATTTATACTTGAACAATTG
54H_trip_rev	CAATTGTTCAAGTATAAATgcAATTATTgcTTTTATGACCgcTTCCGGAACAGATGAATC
54H_D582A_fwd	GTACTGGAAGATGACCGAGcTAAAATCTGTGGCTATCTATG
54H_D582A_rev	CATAGATAGCCACAGATTTTAgCTCGGTCATCTTCCAGTAC
54H_D580A_fwd	GTATATTTACGTACTGGAAGATGcCCGAGATAAAATCTGTGG
54H_D580A_rev	CCACAGATTTTATCTCGGgCATCTTCCAGTACGTAAATATAC
54H_Dub_fwd	GTATATTTACGTACTGGAAGATGcCCGAGcTAAAATCTGTGGCTATC
54H_Dub_rev	GATAGCCACAGATTTTAgCTCGGgCATCTTCCAGTACGTAAATATAC

References:

1. Kunishima, N, A., Y, RIKEN Structural Genomics/Proteomics Initiative (RSGI). Crystal structure of PH1933 from Pyrococcus horikoshii OT3. https://doi.org/10.2210/pdb1wwz/pdb (2005).
2. Rao, F. V. *et al.* Structure of a bacterial putative acetyltransferase defines the fold of the human O-GlcNAcase C-terminal domain. *Open Biol* **3**, 130021 (2013).
3. He, Y., Roth, C., Turkenburg, J. P. & Davies, G. J. Three-dimensional structure of a Streptomyces sviceus GNAT acetyltransferase with similarity to the C-terminal domain of the human GH84 O-GlcNAcase. *Acta Crystallogr D Biol Crystallogr* **70**, 186–195 (2014).
4. Yuan, H., Ferenbach, A. T. & Van Aalten, D. A triple fluorescence approach to measure O-GlcNAc dyshomeostasis in stem cells. Preprint at https://doi.org/10.1101/2025.07.12.664510 (2025).

Uncropped SDS-PAGE gel from Supplemental Figure 1a

The uncropped SDS-PAGE gel contains extra lanes (right-hand side) loaded with a quimeric pHAT mutant unrelated to the experiments reported here. These lanes were not used for data analysis in this manuscript and are therefore not shown in the cropped figure.

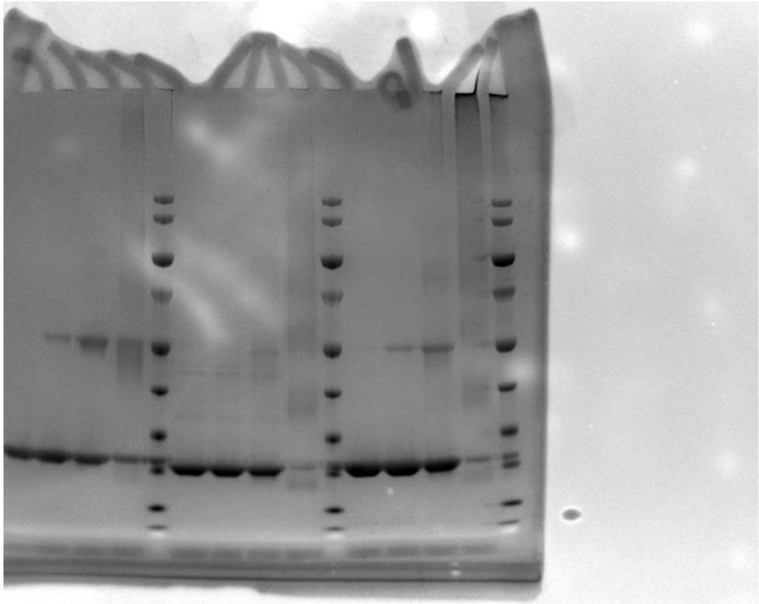

Supplement: Supplementary file 1 — Supplementary Information [file 41467_2025_63893_MOESM1_ESM.pdf]
