# Supplementary material for: Multi-domain O-GlcNAcase structures reveal allosteric regulatory mechanisms
Source: Nat Commun. 2025 Oct 3;16:8828. doi: 10.1038/s41467-025-63893-2 (PMC12494797; doi:10.1038/s41467-025-63893-2)
Supplement: Supplementary file 2 — Reporting Summary [file 41467_2025_63893_MOESM2_ESM.pdf]

nature portfolio

Corresponding author(s): Daan M.F. van Aalten

Last updated by author(s): Aug 21, 2025

Reporting Summary

Nature Portfolio wishes to improve the reproducibility of the work that we publish. This form provides structure for consistency and transparency in reporting. For further information on Nature Portfolio policies, see our Editorial Policies and the Editorial Policy Checklist.

Statistics

For all statistical analyses, confirm that the following items are present in the figure legend, table legend, main text, or Methods section.

n/a	Confirmed	
☐	☒	The exact sample size (*n*) for each experimental group/condition, given as a discrete number and unit of measurement
☐	☒	A statement on whether measurements were taken from distinct samples or whether the same sample was measured repeatedly
☐	☒	The statistical test(s) used AND whether they are one- or two-sided<br>*Only common tests should be described solely by name; describe more complex techniques in the Methods section.*
☐	☒	A description of all covariates tested
☐	☒	A description of any assumptions or corrections, such as tests of normality and adjustment for multiple comparisons
☐	☒	A full description of the statistical parameters including central tendency (e.g. means) or other basic estimates (e.g. regression coefficient) AND variation (e.g. standard deviation) or associated estimates of uncertainty (e.g. confidence intervals)
☐	☒	For null hypothesis testing, the test statistic (e.g. *F*, *t*, *r*) with confidence intervals, effect sizes, degrees of freedom and *P* value noted<br>*Give P values as exact values whenever suitable.*
☐	☒	For Bayesian analysis, information on the choice of priors and Markov chain Monte Carlo settings
☐	☒	For hierarchical and complex designs, identification of the appropriate level for tests and full reporting of outcomes
☒	☐	Estimates of effect sizes (e.g. Cohen's *d*, Pearson's *r*), indicating how they were calculated

Our web collection on statistics for biologists contains articles on many of the points above.

Software and code

Policy information about availability of computer code

Data collection	Flow cytometry: NovoExpress (v. 1.6.2, Agilent, Santa Clara, CA), Crystallography: MXCuBE, CryoEM: EPU, SAXS:Difrac.saxs, SPR: (BioLogic)
Data analysis	Flow cytometry: Analysis: FlowJo(10.10), Structural Biology:XDS, MOLREP, REFMAC5, COOT(0.9.8),Phenix-dev (4788), CryoSPARC (4.6.2) SAXS: ATSAS (4.0.1-1), SPR (BioLogic) Structural data representation:PyMOL (3.1.3), ChimeraX (1.5)

For manuscripts utilizing custom algorithms or software that are central to the research but not yet described in published literature, software must be made available to editors and reviewers. We strongly encourage code deposition in a community repository (e.g. GitHub). See the Nature Portfolio guidelines for submitting code & software for further information.

Data

Policy information about availability of data

All manuscripts must include a data availability statement. This statement should provide the following information, where applicable:

- Accession codes, unique identifiers, or web links for publicly available datasets
- A description of any restrictions on data availability
- For clinical datasets or third party data, please ensure that the statement adheres to our policy

The diffraction dataset together with the atomic coordinates for TapHAT has been deposited in the Protein Data Bank (PDB) under the code: 6R45 [https://doi.org/10.2210/pdb6R45/pdb]. The atomic coordinates for the cryo-EM structures have been deposited under the PDB accession codes: 9QEP (TaOGA) [https://doi.org/10.2210/pdb9QEP/pdb] and 9QEN (hOGA) [https://doi.org/10.2210/pdb9QEN/pdb]. The electron microscopy density maps have been deposited in the Electron Microscopy Data Bank (EMBD) under the accession codes: EMD-53082 (TaOGA) [https://www.ebi.ac.uk/pdbe/entry/emdb/EMD-53082] and EMD-53081

(hOGA) [https://www.ebi.ac.uk/pdbe/entry/emdb/EMD-53081]. The SAXS data have been deposited at the Small Angle Scattering Biological Data Bank (SASBDB) under the accession code SASDXF3 [https://www.sasbdb.org/data/SASDXF3/]. Composite maps and model of conformation I-IV, as well as the two AlphaFold models used in this study, are found as supplementary files.
In addition, the already published atomic coordinates PDB 7VVU [https://doi.org/10.2210/pdb7VVU/pdb], PDB 3ZJ0 [https://doi.org/10.2210/pdb3ZJ0/pdb], PDB 4BMH [https://doi.org/10.2210/pdb4BMH/pdb], PDB 1WWZ [https://doi.org/10.2210/pdb1WWZ/pdb], PDB 1Q2D [https://doi.org/10.2210/pdb1Q2D/pdb] and PDB 5VVO [https://doi.org/10.2210/pdb5VVO /pdb] were used in this study.
Size-exclusion chromatograms, DSF, FACS and SPR raw data files presented in this study can be downloaded as a source data file. Unless otherwise stated, all data supporting the results of this study can be found in the article, supplementary, and source data files. Source Data are provided with this paper.

Research involving human participants, their data, or biological material

Policy information about studies with human participants or human data. See also policy information about sex, gender (identity/presentation), and sexual orientation and race, ethnicity and racism.

Reporting on sex and gender	*Use the terms sex (biological attribute) and gender (shaped by social and cultural circumstances) carefully in order to avoid confusing both terms. Indicate if findings apply to only one sex or gender; describe whether sex and gender were considered in study design; whether sex and/or gender was determined based on self-reporting or assigned and methods used.* *Provide in the source data disaggregated sex and gender data, where this information has been collected, and if consent has been obtained for sharing of individual-level data; provide overall numbers in this Reporting Summary. Please state if this information has not been collected.* *Report sex- and gender-based analyses where performed, justify reasons for lack of sex- and gender-based analysis.*
Reporting on race, ethnicity, or other socially relevant groupings	*Please specify the socially constructed or socially relevant categorization variable(s) used in your manuscript and explain why they were used. Please note that such variables should not be used as proxies for other socially constructed/relevant variables (for example, race or ethnicity should not be used as a proxy for socioeconomic status).* *Provide clear definitions of the relevant terms used, how they were provided (by the participants/respondents, the researchers, or third parties), and the method(s) used to classify people into the different categories (e.g. self-report, census or administrative data, social media data, etc.)* *Please provide details about how you controlled for confounding variables in your analyses.*
Population characteristics	*Describe the covariate-relevant population characteristics of the human research participants (e.g. age, genotypic information, past and current diagnosis and treatment categories). If you filled out the behavioural & social sciences study design questions and have nothing to add here, write "See above."*
Recruitment	*Describe how participants were recruited. Outline any potential self-selection bias or other biases that may be present and how these are likely to impact results.*
Ethics oversight	*Identify the organization(s) that approved the study protocol.*

Note that full information on the approval of the study protocol must also be provided in the manuscript.

Field-specific reporting

Please select the one below that is the best fit for your research. If you are not sure, read the appropriate sections before making your selection.

[x] Life sciences [ ] Behavioural & social sciences [ ] Ecological, evolutionary & environmental sciences

For a reference copy of the document with all sections, see nature.com/documents/nr-reporting-summary-flat.pdf

Life sciences study design

All studies must disclose on these points even when the disclosure is negative.

Sample size	Flowcytometry: at least 30,000 mTagBFP2 positive cells were collected per sample for analysis.
Data exclusions	Not Applicable
Replication	Flow cytometry analysis was performed on four independent biological replicates, with each variant transfected into cells of different passage numbers on separate days.
Randomization	Not Applicable
Blinding	Not Applicable

Reporting for specific materials, systems and methods

We require information from authors about some types of materials, experimental systems and methods used in many studies. Here, indicate whether each material, system or method listed is relevant to your study. If you are not sure if a list item applies to your research, read the appropriate section before selecting a response.

Materials & experimental systems

n/a	Involved in the study
☐	☒ Antibodies
☐	☒ Eukaryotic cell lines
☒	☐ Palaeontology and archaeology
☒	☐ Animals and other organisms
☒	☐ Clinical data
☒	☐ Dual use research of concern
☒	☐ Plants

Methods

n/a	Involved in the study
☒	☐ ChIP-seq
☐	☒ Flow cytometry
☒	☐ MRI-based neuroimaging

Antibodies

Antibodies used	RL2-Alexfluor647 (Thermo)
Validation	This RL2 monoclonal antibody recognizes proteins with O-N-acetylglucosamine (O-GlcNAc) glycosylation. It was originally developed by immunizing mice with the rat liver nuclear envelopes containing nuclear pore complexes. The RL2 clone has been successfully used in Western blot, immunofluorescence, immunoprecipitation, and flow cytometry in a wide variety of mammalian cells.

Eukaryotic cell lines

Policy information about cell lines and Sex and Gender in Research

Cell line source(s)	The cell line used in this study is derived from E14-TG2a.IV (129/Ola) ES cells.
Authentication	Not authenticated
Mycoplasma contamination	Cell line tested negative for mycoplasma contamination
Commonly misidentified lines (See ICLAC register)	*Name any commonly misidentified cell lines used in the study and provide a rationale for their use.*

Plants

Seed stocks	*Report on the source of all seed stocks or other plant material used. If applicable, state the seed stock centre and catalogue number. If plant specimens were collected from the field, describe the collection location, date and sampling procedures.*
Novel plant genotypes	*Describe the methods by which all novel plant genotypes were produced. This includes those generated by transgenic approaches, gene editing, chemical/radiation-based mutagenesis and hybridization. For transgenic lines, describe the transformation method, the number of independent lines analyzed and the generation upon which experiments were performed. For gene-edited lines, describe the editor used, the endogenous sequence targeted for editing, the targeting guide RNA sequence (if applicable) and how the editor was applied.*
Authentication	*Describe any authentication procedures for each seed stock used or novel genotype generated. Describe any experiments used to assess the effect of a mutation and, where applicable, how potential secondary effects (e.g. second site T-DNA insertions, mosiacism, off-target gene editing) were examined.*

Flow Cytometry

Plots

Confirm that:

☒ The axis labels state the marker and fluorochrome used (e.g. CD4-FITC).

☒ The axis scales are clearly visible. Include numbers along axes only for bottom left plot of group (a 'group' is an analysis of identical markers).

☒ All plots are contour plots with outliers or pseudocolor plots.

☒ A numerical value for number of cells or percentage (with statistics) is provided.

Methodology

Sample preparation	Established mouse embryonic stem cell line where endogenous OGT and OGA are CRISPR tagged with fluorescent marker were used for transfection experiments
Instrument	NovoCyte Quanteon 4025 flow cytometer equipped with four lasers (405 nm, 488 nm, 561 nm and 637 nm) and 25 fluorescence detectors (Agilent, Santa Clara, CA).
Software	Software acquisition: NovoExpress (v. 1.6.2, Agilent, Santa Clara, CA).

	Analysis: FlowJo(10.10).
Cell population abundance	Cells were analysed by flowcytometer without cell sorting
Gating strategy	Cells were analysed using forward scatter height (FSC-H) and side scatter height (SSC-H) to distinguish cells from debris. Gated cells were then assessed using FSC-A vs. FSC-H and SSC-A vs. SSC-H plots to identify singlet populations. The gated singlet cells were subsequently analysed using mTagBFP2-A vs. RedDot2-A to distinguish live, transfected mTagBFP2-positive cells (the mTagBFP2-positive cells were gated in comparison with untransfected control). Finally, the gated transfected cells were plotted in histograms for OGT-sfGFP and mScarlet3-OGA, and the median fluorescence intensity from each plot was extracted for statistical analysis.

☒ Tick this box to confirm that a figure exemplifying the gating strategy is provided in the Supplementary Information.
